# The Hospital. Nursing Section

**Published:** 1905-03-18

**Authors:** 


### The Hospital.

**Nursing Section.**

Contributions for this Section of "The Hospital" should be addressed to the Editor, "The Hospital," Nursing Section, 28 & 29 Southampton Street, Strand, London, W.C.

No. 964.—Vol. XXXVII. *SATURDAY, MARCH 18, 1905.*

### Notes on News from the Nursing World.

### A ROYAL SURPRISE VISIT.

On Tuesday afternoon, shortly before four o'clock, the nursing staff at St. Mary's Hospital, Paddington, were excited by the quite unexpected appearance of the Prince of Wales. His Royal Highness, in his capacity of President of the hospital, had come to pay the institution a surprise visit. The Prince drove up in a private brougham, attended by an equerry, and remained for upwards of an hour. The matron accompanied him round the wards, and answered his inquiries.

### THE HIGHER EDUCATION OF NURSES.

In the House of Commons on Monday, Mr. Gerald Balfour, answering Mr. Healy, said: "An application has been received by the Board of Trade for a license to incorporate, under Section 23 of the Companies Act, 1867, without the word 'limited,' a society for the higher education of nurses. The proposed memorandum and articles of association have been submitted to the Board of Trade, and the Board propose to hear both the applicants and the objectors to the grant of a license. Until after such hearing they do not propose to come to any decision as to policy or otherwise." The President of the Board of Trade had previously declined to receive a deputation on the subject of the application of the Society. The request was, no doubt, made in ignorance, for—as it was pointed out in the official reply—the Society in question having been instructed to advertise the application in the usual way, and it being proposed in due course to hold an inquiry, at which those who have made objections to the applications will be given an opportunity of stating the grounds of their opposition in the presence of representatives of the applicants, there was obviously no reason why the President should make an exception to the ordinary custom in such cases.

### THE LEGACY OF MR. LONG.

The fact that Mr. Walter Long has become Chief Secretary for Ireland, and was succeeded as President of the Local Government Board by Mr. Gerald Balfour, is not without interest to nurses, especially to those who are engaged in Poor-law work. What is now the position in respect to the report of the departmental committee? Mr. Long has gone out of office at Whitehall without issuing an order, and it is open to Mr. Gerald Balfour to ignore his legacy. It will none the less be a public scandal, as well as a slap in the face to the advocates of reform in Poor-law nursing if, after all the time and all the money which were spent in pursuit of the inquiry by the departmental committee, their report is treated as waste-paper.

### THE ACLAND HOMES.

It was decided at the annual meeting of the subscribers to the Sarah Acland Memorial District Nurses' Home and the Acland Nursing Home to affiliate the former to Queen Victoria's Jubilee Institute. In face of this fact certain criticisms by a former treasurer of the Homes on the report submitted at the meeting, do not seem to us to need notice. It will be generally hoped that under the new conditions the District Nurses' Home will be as useful as it has been in the past, and that there will be a sufficient increase in the financial support accorded to it to enable the committee to maintain enough nurses to meet the requirements of the sick poor. There is no doubt, however, that the Acland Homes, like other institutions, have suffered from the combination of district and private nursing. Because of the latter many people have thought that the Homes were managed entirely on business lines and paid, or ought to pay, their own way; and because of the former, it has been feared that fees earned by the private nurses may have been unjustifiably used in the cause of charity. We are glad that Mr. H. Acland suggested that a statement of the accounts between the Nursing Home and the District Nurses' Fund should be published after an investigation from the beginning, so that the precise situation may be rendered perfectly clear to every one.

### THE VALUE OF WRITTEN AGREEMENTS.

A case came before the Marylebone Court on Monday in which a nurse sued her sister and another lady for £12, which she claimed was due to her as salary. The defendants had entered into a deed of partnership to carry on together a nursing home in Marylebone, engaging the plaintiff to assist them in the conduct of the home at a salary of £25 for the first year. This was in April last year; but it took longer than was anticipated to get the home prepared, and it was not ready for the reception of patients until September. During the interval the plaintiff helped to select furniture and wrote business letters for the defendants. The home was not a success, and the partnership was dissolved the following November. The defendants maintained that, as the business of the home did not commence until September, the plaintiff was only entitled to salary for that time, even though she had assisted previously in other ways. The judge, however, ruled that as the deed provided that there "shall be a partnership from the date hereof," which was April 28th, and as the plaintiff's engagement was to commence with the partnership, judgment must be in her favour for the amount claimed, with costs. We have often urged upon nurses the need of being more business-like and of reducing to writing any arrangement into which they may wish to enter. Here they can see for themselves the wisdom of our advice. Had there been no written document to fall back upon the plaintiff would probably have lost her case.

### QUEEN'S NURSES AND THE POOR RATE.

A REMARKABLE proof of the value of the work of the Queen's Nurses from the standpoint of the over-burdened ratepayers is afforded by a statement made at the annual meeting of the supporters of the Torquay Nurses' Institution, which is a branch of Queen Victoria's Jubilee Institute. Dr. Makeig Jones, in an address describing the work of the Queen's Nurses, said that the number of old persons who were kept out of the workhouse in the four districts visited by them caused a saving to the poor rate of £720 a year. If only out of gratitude for such a reduction as this, which exceeds in amount the total sum required to meet the expenses of the Nurses' Institution, the Torquay people should subscribe liberally to that organisation.

### PRAYERS IN SICK-WARDS.

AT the last meeting of the Chapel-en-le-Frith Guardians, a rule, authorising the charge nurse to read prayers every night to the inmates of the infirmary was discussed at some length. Father Hayward objected on principle to any prayers or religious services being conducted in a place from which the inmates could not get away, even if they wanted to do so, and moved the rejection of the rule. The motion having been put to the vote, there were 18 in favour of the charge nurse reading prayers and six against. We recognise the force of Father Hayward's plea, and we admit that the spiritual needs of the patients can best be met by individual ministration on the part of the chaplain to those who desire to receive them. But we scarcely think that the adoption of the rule will give rise to a religious grievance.

### SUBSTANTIAL DAMAGES FOR A NURSE.

AT the Swindon County Court last week a nurse brought an action against her employer for breach of engagement, for assault and detention of property, and railway fares. The plaintiff had been engaged as nurse-companion and secretary at a salary of £1 1s. a week with board and lodging, the defendant, who suffered from gout and rheumatism, agreeing to pay her travelling expenses from Stonehouse to Marlborough. It was alleged that two days later in the evening the defendant used threats and violence towards the plaintiff, shouted "I will kill you," attempted to seize her by her hair, and pushed the door upon her. As the result of his threats, plaintiff was obliged to leave the defendant's house. He then refused to deliver up to the plaintiff her wearing apparel, letters, and chattels, and in consequence of not receiving her letters she was unable to secure other professional engagements. When the plaintiff went to claim her belongings she found her bicycle half buried in the garden, and decorated with flowers. The housekeeper to plaintiff who had been with him for 14 years, admitted that he took too much drink, and that he had threatened her also, but said that she "always went out when he got into a temper." No evidence was called for the defence, and the jury awarded the plaintiff £28 16s. damages, and costs. This verdict appears to us to be very proper, and we hope that it will have the effect of warning defendant and others of his class that because a nurse is a woman, he is not at liberty to treat her with abominable rudeness.

### PETTY DISPUTE AT BRIDGWATER.

THE officers at Bridgwater Workhouse and Hospital appear to find it difficult to live happily together. At the fortnightly board meeting one of the matters for the consideration of the Guardians was a complaint made by the head nurse that the laundress had assaulted her in the kitchen. The nurse stated that she had been struck on the chest and shaken, whilst the laundress maintained that she simply took the nurse by the shoulder. But the real grievance seems to be that the washing of the hospital linen is now done at the workhouse, and the laundress resents the great number of things which the nurse sends to be cleansed, there being sometimes as many as 107 sheets in one day for 70 patients. In the course of her evidence, the nurse admitted that she had made no complaint of the assault to the master or matron, because "she did not expect to get their sympathy, so preferred to bring the matter straight to the board." Finally, the chairman called in the nurse, bade her remember that the master and matron were the heads of the institution, and that all complaints for the future must go to them first. If they did nothing in the matter the Guardians would then take it up. The laundress was warned that even under great provocation no officer was allowed to assault another officer. Under a proper system such incidents as this would not be possible.

### A QUESTION OF TITLE.

AT the annual meeting of the Birmingham District Nursing Society one of the speakers suggested that its name was one of the causes of the heavy deficit in the working in 1904, which amounted to £388. He maintained that the public could more readily understand that the society was not kept by the guardians or in rates if the name did not include the word "District." We are glad to learn that the suggestion was not adopted. As it was urged in reply, the use of the word "District" is official and universal, and we certainly prefer the title of Birmingham District Nursing Society to that of Birmingham Home Nursing Society. But it now, more than ever, behoves those who take this view in Birmingham to work hard during the current year in order that the organisation may be put on a proper financial basis, and be able at an early date to send out nurses to the newly-built district at the extreme city boundary.

### THE VICTORIAN ORDER OF NURSES.

THE proceedings at the annual meeting of the Toronto branch of the Victorian Order of the Nurses indicate that the movement is greatly appreciated. In the course of his speech the honorary treasurer paid a tribute to the order and said that it was now known from the Atlantic to the Pacific, its nurses being in great demand everywhere. He mentioned that Lady Minto collected an endowment of 110,000 dollars for cottage hospitals in connection with the order, and that there are now fourteen of these hospitals, with sixteen nurses scattered over the country. The financial position of the Toronto branch is as sound as the work of the nurses is satisfactory. There was a balance of income over expenditure for 1904, and the number of visits paid by the nurses was 6,330, the number of cases being 499. We learn that Miss Annie Barratt, who for some time was sister at Birmingham Infirmary and night superintendent at Plaistow Hospital, has joined the order and will shortly take up her duties in the North-West district.

### DISTRICT NURSES AND NIGHT WORK.

At the annual meeting of the Cambridge District Nursing Association one of the speakers suggested that the organisation should undertake night nursing. We presume that, in cases of emergency, a district nurse would remain with a patient during the night. In ordinary circumstances this, of course, is not essential, and if systematic, night nursing is to be undertaken by the district nurses at Cambridge, the increase of the staff will become an immediate necessity. At present the district of Chesterton is still without a nurse, the aid offered by the Board of Guardians being inadequate, and it is therefore evident that additional financial support is needed before further developments can be decided upon. During last year, with a staff of seven, including the lady superintendent and one assistant, the nurses attended 1,274 cases, and paid 34,137 regular and 896 casual visits. They cannot be expected to do more.

### QUEEN'S NURSES AT THE MANSION HOUSE.

There was nothing unusual in the proceedings on Tuesday afternoon at the annual meeting of the East London Nursing Society, whose annual report we dealt with last week. The Lord Mayor in his opening speech warmly praised the work of the Queen's Nurses amongst the poor in East London, and emphasised the appeal for further assistance. Other speakers at the Mansion House included Mr. Alderman Strong, the Rector of Bow, Sir E. Hay Currie, and Sir W. Quayle Jones. As on former occasions, the matrons and nurses belonging to the society were present.

### A JOURNAL FOR CANADIAN NURSES.

We have received from Toronto a copy of the first number of *The Canadian Nurse*, a quarterly journal for the nursing profession in Canada. It is edited by Dr. Helen McMurchy, contains a variety of interesting matter, including an instalment of an account of the Toronto General Hospital Training School for Nurses by Miss Agnes Snively, with her portrait, a contribution by Mrs. Hampton-Robb, and a careful selection of news from the nursing world in the Dominion.

### EFFECT OF ECONOMY AT DARWEN.

A year ago, at the annual meeting of the Darwen District Nursing Association, Mrs. C. P. Huntington, the president and founder, urged the need of economy in the administration of the funds. Her advice seems to have been taken to heart, for it was announced at the annual meeting that, after the payment of all expenses during 1904, the Association has in hand a balance of £111, as against £39 on January 1st last year. This is the more a matter for congratulation because the Nurses' Home at Darwen does not provide adequate accommodation for the increasing staff, and it will shortly be essential to make a move with the view of extending it. So much enthusiasm was, however, shown at the meeting, and there is such a strong committee of ladies that it is not likely that there will be any serious difficulty with regard to the comfortable housing of the Queen's Nurses.

### PROSPERITY AT ST. HELENS.

The satisfactory report of the St. Helens District Nursing Association, which was the subject of congratulation by the speakers at the annual meeting, affords another indication of the admirable manner in which the branches of the Queen's Jubilee Institute are managed. We observe, again and again, that while nursing organisations in the country which are run on what are called independent lines have frequently a considerable deficit, those which are affiliated to the Institute have often a substantial balance to the good. At St. Helens the income for 1904 exceeded the expenditure to the extent of £145, and though the balance will be required for a new district, the fact remains that the association is in a perfectly prosperous financial condition. We notice that, in response to an appeal for support in the works of the town, the association received £52 from the workpeople as well as subscriptions and donations from several firms. It has now been established 20 years, and is appreciated by all classes.

### DEFICIT AT HASTINGS.

At the annual meeting of the Hastings District Nursing Association, attention was called by Lord Brassey, who presided, to the fact that though the record of the work of the nurse is highly satisfactory, showing that during 1904 the visits paid were 12,851 as compared with 9,824 in 1903, the expenditure exceeded the income to the extent of £135. Moreover, it was stated that the excess would have been greater if it had been requisite to pay the lady superintendent for a full year instead of only for six months. It is encouraging that the income was considerably more than in 1903, and we anticipate that if the collectors are sufficiently energetic they will obtain this year a sum large enough to cover the needs of the organisation. Of the 198 cases in the maternity department no fewer than 62 were attended under the provisions of the Midwives Act.

### SHORT ITEMS.

At the last meeting of the Ipswich Nurses' Home Committee a letter was read from the executors of the late Mr. J. E. Ransome, stating that Mr. Ransome had left a legacy of £500 to the Home. A resolution expressing thankfulness for the much-needed gift was passed.—The appointment of Miss H. M. Drage and Miss L. M. Toller as staff nurses in Queen Alexandra's Imperial Military Nursing Service has been confirmed, and Miss G. S. Wyatt has resigned the post of staff nurse.

### The Nursing Outlook.

"From magnanimity, all fear above;
From nobler recompense, above applause,
Which owes to man's short outlook all its charm."

### SELF-HELP AND CO-OPERATION.

The report of the eighteenth annual meeting of the members of the Royal National Pension Fund for Nurses, which we publish this week, cannot fail to inspire feelings of pride amongst the nurses of the Empire. It is not a little remarkable that the army of women workers whom the nurses represent should have accumulated in their Pension Fund in seventeen years nearly one million pounds sterling. No women are more loyal than the nurses, and they will be grateful to her Majesty the Queen for having declared that she considers "the success of this Fund to be entirely satisfactory and remarkable." We have never failed to point out that the Royal National Pension Fund for Nurses belongs to nurses in its entirety and to them alone. Every farthing that the nurses' money can earn under the able administration of the leading financiers of the City of London belongs to and is divided amongst the nurses who are policy-holders in this the largest thrift fund of women workers in the world. His Majesty the King, whose wonderful activity and energy have won for him the love and confidence of his people, has written that "he considers it most creditable on the part of all those who have the management of the Pension Fund that it should have been placed on such a sound foundation, and that its progress during the past year should have been so excellent." This unique testimony to the appreciation which this effort at self-help on the part of the nurses has excited throughout the Empire may cause many nurses everywhere to inquire into the working of the Pension Fund with a view to taking out a policy with as little delay as possible.

We have said that the Royal National Pension Fund belongs to the nurses in its entirety. That is to say, it is a mutual society which has the credit of being the most economically administered of insurance offices in this country, and the consequent additions out of profits, for every penny belongs to the policy-holders, steadily augment the money which each nurse may entrust to its keeping. The excellence of the management reflects the greatest credit on Mr. Louis H. M. Dick, the secretary, and on the gentlemen who constitute the staff working under his guidance. This excellence has excited the gratitude of many policy-holders, and it was their wish, as recently expressed more than once, to in some way mark their sense of indebtedness to the officials of the Fund. This proof of the good feeling of the policy-holders has given unfeigned pleasure to the gentlemen in question. The suggestion could not, however, of course be thought of for a moment, for nurses, as working women, have for the most part to make considerable sacrifices in order to provide for their own future. We learn however from Mr. Dick, that policy-holders could not show him or his staff their appreciation for anything which they may have done for nurses, in any more gratifying way, than by each one trying to do her very best to increase the membership of the Pension Fund, by bringing it more prominently before those nurses who have not yet taken out a policy for their own security. Mr. Dick states that during the last three or four years he has had very much greater insight into the possibilities of the Pension Fund than during the preceding seven or eight years, and he has become more and more convinced, that the membership could easily be doubled, if the existing policy-holders were never to let an opportunity pass of impressing the value of the Fund upon every nurse they may meet who has not yet joined it. Mr. Dick states, further, that to see the number of the policy-holders materially increased would afford him greater satisfaction than anything else in connection with the work for the Fund, for it would mean, that later on, fewer and fewer nurses would be left in comparative poverty.

One nurse, Miss M. D. Brinton, has written a pamphlet entitled *The Pension Fund*, by *An Enquiring Policy-holder*, copies of which may be obtained free on application to the Secretary of the Fund, at 28 Finsbury Pavement, London, E.C. At the annual meeting Miss Brinton was accorded a unanimous vote of thanks for the services she had rendered in writing this pamphlet, and we would counsel every one of our readers, being a nurse, to obtain a copy and to study it carefully. If this course be adopted and every existing policy-holder will make a point of endeavouring during 1905 to explain the advantages of the Fund to at least one nurse who is not a member, and to get her to commence to save by taking out a policy, the results cannot fail to be both remarkable and gratifying. Remarkable in the sense that a large body of women workers, at present unprovided for, will have thus entered a haven of refuge where they can find sustenance and help throughout their lives. And gratifying to the existing policy-holders, who will render this measure of personal service to their fellow workers, from the circumstance, that every recruit so obtained will tend to strengthen the Fund, and so to benefit every member of it. Let nurses then take for their motto "United we stand," and let every policy-holder become zealous in the cause of thrift through the Fund. There is no course which could be suggested which is better calculated to give pleasure to every policy-holder, or to add so much to the benefits which will be likely to accrue to each nurse, for the self-denial which she may have exhibited, by continuing to invest, every year, a portion of her earnings in the Royal National Pension Fund for Nurses.

### Lectures on the Nursing of Sick Children.

By James Burnet, M.A., M.B., M.R.C.P.Edin., Registrar, Royal Hospital for Sick Children; Clinical Tutor, Extramural Wards, Royal Infirmary; and Physician to the Marshall Street Dispensary, Edinburgh.

### II.—THE FEEDING OF SICK CHILDREN.

The subject of our present lecture is one of very great importance to every nurse. As a general rule the physician gives directions for the feeding of his young patients, but he always expects the nurse to carry out the details for herself. It is necessary, therefore, that the nurse should know sufficient about the feeding of sick babies and young children so that she may be, to a certain extent, self-reliant. In private work more especially, perhaps, the nurse who knows how to feed her sick charges will be a favourite both with medical practitioners and with the public who employ her. We feel that one lecture is all too short for the consideration that this subject deserves, but we shall endeavour to compress into it as many useful facts as we can.

### Some General Rules to be Observed in the Feeding of Infants who are Ill.

When a baby's temperature rises above the normal its powers of digestion become impaired. Not uncommonly vomiting occurs, and in many cases he refuses all food. His thirst, however, becomes correspondingly greater, and this is a point which must never be forgotten. The baby cannot tell you he is thirsty, but his wishes must be anticipated by giving him a sufficiency of plain cold water at regular intervals. Remember, therefore, that *when the thermometer rises you must supply the patient with water.* At the same time *the amount of food must be reduced.* We may have to give only one-half of what the baby was getting while in health; in other words, we must dilute his food so that it will be more easy of digestion.

In some cases it may become necessary to *peptonise the food* so as to render it more or less predigested and ready of assimilation. A very good method of carrying this out is to use peptogenic milk powder. A teaspoonful of this is added to every bottle of food. After mixing thoroughly, the whole is heated gently for about six minutes, stirring all the time. It should then be cooled to the temperature of the body before being given to the patient.

Instead of ordinary cow's milk the physician may order *condensed milk.* This is usually given in too great strength. It should never be given less dilute than one teaspoonful of the milk to three or four tablespoonfuls of water. This often does well when ordinary cow's milk is vomited. Another substitute for cow's milk is *whey,* which is really milk without the curd. Whey may be readily prepared by heating a pint of milk and then slowly adding two teaspoonfuls of essence of rennet. The mixture is then to be set aside for about 20 minutes until firm clotting has taken place. The curd is then separated and the liquid whey strained off.

Sometimes *barley water* is used for diluting the milk mixture. This can be quickly prepared by the nurse from ordinary pearl barley. The latter must first be thoroughly washed, and thereafter a dessertspoonful is placed in a pint of cold water in a pan. This is boiled down to about two-thirds of a pint and then strained. If the mixture is kept boiling too long barley jelly will be formed, which is much too thick for mixing with milk. As a stimulant and nutrient *sherry whey* will be found useful. To prepare this the nurse should take about half a pint of milk and add to it a wine-glassful of sherry. This should then be boiled, allowed to cool, and the liquor strained off.

### The Feeding of Older Children.

In the case of older children the digestive disturbance is not likely to prove so troublesome as in young babies who are ill. *The mistake usually made here is to give the child nothing but milk.* Now it should be remembered that milk, when given alone, makes the mouth dry, increases the thirst, and very often causes flatulence and constipation. Milk is a food, no doubt, but a sick child may get too much of it. It is usually best given diluted with lime-water, and between times Vichy or soda-water may be given to drink. It is *a mistake to force a child to take food* when he is ill, especially if he does not seem to care for it. It is astonishing how little food is necessary to keep a sick child going provided he is given plenty of fluid. Instead of milk it is usually advisable, unless countermanded by the physician, to give such things as beef-tea, broth, raw eggs, and koumiss. Often a little orange or grape-juice proves very pleasing to a sick and restless child.

### Rectal Feeding and How to Carry it Out.

In some cases of illness it may be advisable to feed the child by the bowel. The attendant usually says, "Nurse, give the child a nutrient every four hours"; that is all, and she is left to find out what to give and how to give it. I shall try to give some information on this matter. The first thing to be done is to thoroughly *wash out the bowel with plain hot water.* If this is not done the nutrient enema will be of no value whatever. After the washing-out process has been completed the child should be allowed to *rest for at least half an hour,* and then he should be turned on his back with knees well drawn up and the hips raised by placing a pillow under them. By using these precautions there will be a greater chance of the enema being retained. The points to attend to in giving a nutrient enema are to *give it slowly,* and to *give it in small quantity,* not more than about five tablespoonfuls (preferably less) at one time. After it has been administered the hips are still to be kept raised for some time, and held together. We prefer to use a large glass syringe with a piece of rubber tubing attached for rectal feeding, as it is easily kept clean and is handy to use.

Now as to what to give. The enema we usually order is made up as follows:—Milk, three tablespoonfuls; cream, one dessertspoonful; yolk of egg. These ingredients are heated together and then allowed to cool. It is usually advisable to add a full teaspoonful of liquor pancreaticus to the mixture while being heated.

Peptonised milk or gruel may also be given by the rectum, while *suppositories* containing meat extract and milk can be obtained from the chemist.

Nasal Feeding: Its Difficulties and How to Overcome Them.

This method of feeding is not often ordered, but every nurse should know how it is carried out. The patient must first be rolled in a blanket so that he cannot possibly struggle, and placed on his back. The end of the tube should be well oiled and then directed straight backwards, not upwards, along one nostril parallel with the roof of the mouth. It should then be gently guided inwards for about 8 or 9 inches, when it will be found to have entered the stomach if the patient is an infant, but if older it may be necessary to push it in for fully 3 inches more. *Before introducing the food a little plain water should be poured down the tube* so as to make sure that it has gone the right way. Any liquid food may be given by the nasal tube. When the feeding process is completed the tube should be withdrawn slowly and the child allowed to rest quietly. The *tube may pass into the mouth* instead of going down into the stomach, and *it may even get into the larynx.* It is well, therefore, to look into the mouth before starting to feed the patient. If the tube has got into the larynx, which is rarely found to be the case, a fit of coughing will be induced when the tube must be instantly withdrawn and reintroduced.

In conclusion let me say that the secret of the successful feeding of sick children lies in the *avoidance of over-feeding* and in seeing that *regularity* is observed. As a rule an interval of three hours between each meal is the shortest that should be allowed, and between times *abundance of water* should be supplied whether the child asks for it or not.

### The Royal National Pension Fund for Nurses.

The 18th annual general meeting of the members of the Royal National Pension Fund for Nurses was held at River Plate House, Finsbury Circus, on Thursday, March 9, 1905, under the presidency of Sir Henry Burdett, K.C.B. Supporting the chairman were the following members of Council:—Mr. Thomas C. Dewey, F.I.A., Mr. Thomas Bryant, F.R.C.S., Mr. C. Eric Hambro, M.P., Mr. Walter S. M. Burns, Mr. R. Ernest Alexander, Dr. H. P. Hawkins, Dr. S. H. Habershon, and Mr. Falconer L. Wallace.

Amongst those present were:—The Hon. Herbert C. Gibbs, Mr. Alfred J. Waley, Dr. George W. Potter, Mr. Perceval A. Nairne (Chairman, Seamen's Hospital, Greenwich), Mrs. Pritchard Binnie (Hon. Secretary, Junius S. Morgan Benevolent Fund), Mrs. Bretland Farmer (Secretary, Junius S. Morgan Benevolent Fund), Miss Swift (Matron, Guy's Hospital), Miss Oxford (Lady Superintendent, Guy's Trained Nurses' Institution), Miss Smedley (Matron, St. George's Hospital), Mr. Charles Cutting (Royal Free Hospital), Miss Laurence (Matron, Nurses' Home, Salisbury), and a very large number of policy-holders.

The Secretary (Mr. Louis H. M. Dick), read the notice convening the meeting, and an abstract from a letter from Mr. King, in which he says:—"I beg of you to express to the meeting my great regret at being absent, and to assure them that it would have given me great pleasure to attend, and to express personally my appreciation of the extremely prosperous condition of the Fund, and of the excellent progress that is being made in the direction of developing its usefulness."

A Prosperous Year.

Sir Henry Burdett, in addressing the meeting, said:—It gives me very great pleasure to meet the nurses this year, especially as illness prevented me from being present at the last annual meeting—the first meeting since the establishment of the Fund which I was unable to attend. We have had a very prosperous year, and the Fund is extending in all directions satisfactorily. For instance, in 1902 the actual policies issued were 869, and they increased in 1904 to 1,185. The surrenders amounted to 414 in 1902, and to 425 in 1904, and the actual money paid on account of surrenders was £22,736 in 1902, and £23,747—or £1,000 more—in 1904. These surrenders represent a very important side of this Fund, amounting, in fact, to a savings bank, because it is perfectly true, as most nurses know, that if it had not been for the Pension Fund, not one single pound of that money, in all probability, would ever have been put by, and would never have been drawn out in the large sum mentioned, that the nurses might start in business, get married, or do a great many other things which they consider highly desirable, and, I hope, entirely pleasant. (Laughter.) The withdrawals, therefore, are not unsatisfactory from our point of view, for the nurses were put in the position, in their way, of being small capitalists. There is another point, and a very good one, about the surrenders—namely, that we find that as the number of the policy-holders goes on steadily increasing year by year, the percentage of surrenders to the total policy-holders in the Fund decreases; that is to say, there is a tendency year by year amongst those who come into the Fund—while some who came into it have disappeared—to exhibit a steadfast belief in the policy of thrift, and to continue to pursue it throughout their lives. Coming to the funds, the money that we have actually got, I have much pleasure in stating that the total amount at December 31st, 1904, was £931,064, so that if we live another year, this Pension Fund within 20 years of its existence will have accumulated funds exceeding one million sterling.

The Office Staff.

I think I may say here, in passing, that we of the Council are very conscious of our indebtedness to the staff, and especially to Mr. Dick, the secretary. This Fund could never have attained the strength and dimensions it has done if the organisation of the office had not been thorough, and if we had not had some one at the head of it who was really more than an ordinary business man, doing his ordinary business thoroughly well in a business way. Indeed, in the secretary we have a man to whom the work is of vital interest, daily interest, absorbing interest, and in that way his very enthusiasm on behalf of the cause of thrift amongst nurses has been an actual force in itself in attracting a number of policy-holders to come in and join the Fund.

The Investments.

In referring to the investments of the Fund Sir Henry Burdett said:—I do certainly think that the members of the Investments Committee, especially those gentlemen in the City who are at the head of the financial business, who give such a large amount of individual time and attention

ROYAL NATIONAL PENSION FUND FOR NURSES—*Continued.*

on behalf of the nurses in looking after their money, are to be congratulated and thanked that it should be possible to say, in times like these, that the whole investments of the Fund, deducting the income-tax, yielded an average return for 1904 of £4 1s. 3d. per cent. (Cheers.) I am not going to say anything more about the nature of the investments, because we shall have the opportunity of hearing later the report and opinion upon those securities by the gentleman who has investigated them so fully on behalf of the Council for the purposes of the Board of Trade.

### The Importance of Co-operation.

We feel that the nurses have a great opportunity in this Fund of helping their sisters as well as of helping themselves. We can never impress on you too often that this is a mutual society—that is to say, that all the Council are unpaid and act entirely voluntarily, that the expenses, as I have shown you, are cut down to the smallest possible dimensions, and that, in addition to that, every penny of profit which is made in any way goes directly back to the annuitants or the policy-holders in the Fund itself. In a mutual society the greater the number of the members the greater the benefit to the individual members. If therefore every nurse who has a policy in the Pension Fund were to make up her mind that she will testify, as it has been put to me, her appreciation of what is being done for her through this Fund by determining that every year she will help some sister nurse by explaining the work, the objects, and the advantages of the Fund to her and get her to take out a policy, she will be indirectly benefiting herself by increasing the number of policy-holders and directly saving many a nurse from much avoidable misery which she may otherwise suffer from the want of learning how to save. There are a number of people about to-day—a number of nurses in every community of nurses—who maintain that it is of no use trying to save, that it cannot be done, and that they have not enough money to put by, for the burden of it would be utterly impossible to bear. What I want to make the burden of my speech to you to-day is, Don't you believe it. (Laughter and cheers.)

### What Nurses are Doing.

Every woman in the nursing world can save if she likes, and Mr. Dick or myself could give many instances in this Fund of the power of saving. I may mention that 35 out of 40 nurses in a hospital in the West of England are members of the Fund—35 out of 40. Yet, it is said that nurses cannot afford to save? Again, eight out of 10 nurses in a nursing institution, quite a different thing from a hospital, where they are largely dependent on their own exertions, in the South-west of England are policy-holders. That is 80 per cent. These two instances are quite enough, I think, to show you that nurses are not prevented from saving owing to the want of money. There may be want of will on their part or the absence of a friendly policy-holder to explain the Fund and its advantages, and thus to act as a missioner in the cause of thrift; but whatever the reason of a nurse not becoming a policy-holder, in 90 to 95 per cent. of the cases, it cannot be the want of the power to save if she wishes to save. In the case of private nurses (and there are some private nurses present) where the women are somewhat older, the nurse will have all sorts of schemes suggested to her, each of which she will be told is better than that of the Pension Fund—she will be told so, that is, by those who are not members of the Pension Fund—because there are a certain number of nurses who seem to delight in nothing so much as running down the Pension Fund. Consequently, it is the business of those who are members—that is, of the policy-holders—to show the advantages which the Fund gives and not merely suggests, as in other cases. As an example of what can be done, I will quote this fact—that in one London institution 43 out of 60 nurses are members of the Fund, and these nurses hold among them 70 policies. This shows that as time goes on and they are able to collect more money, the nurses immediately invest it in the Pension Fund.

### Help Each Other.

There is one striking instance of the gratitude of nurses which has come out since the report was issued. In the report of the Benevolent Fund the nurses are asked to give 1s. a year. The amounts you have contributed in these shillings have been pretty steady, numbering between 600 and 700 a year; but one nurse this morning sent three £1 Scotch notes from Scotland—a thank-offering for recovery from illness and in appreciation of the benefit she received from the Fund, and she asked the secretary to carry the amount to the Junius S. Morgan Benevolent Fund. (Cheers.) That is an instance which has given great gratification to Lady Rothschild, the hon secretary, and the ladies who administer the Benevolent Fund. It gave great pleasure to me, and I am sure it will give you great pleasure.

### Queen Alexandra's Message.

I do not know how many nurses had the privilege of enjoying that beautiful afternoon at Buckingham Palace last July, but those who were there must have been very much impressed by the power of numbers, and must have appreciated the hospitality and kindness of her Majesty in receiving the nurses, and especially for presenting the certificates to them. (Cheers.) As you know, the Queen is the President of the Fund, and on sending her Majesty the report this year I received a letter back, in which Miss Knollys says: "I lose no time in telling you that I have already submitted your letter and the report, and **I am desired by her Majesty to express her great satisfaction, and to add that she considers the success of this Fund to be entirely satisfactory and remarkable.**"

### His Majesty the King's Opinion.

I have also received a letter from the King, and I appreciate this letter very much, because the King is the Patron of so large a number of institutions that one can hardly expect that he would have time or interest enough to go into the report of the Pension Fund. His Majesty, however, appears to have gone into it fully, for Lord Knollys writes: "I have submitted to the King the secretary's report of the Royal National Pension Fund for 1904, and he desires me to say he has gone through it with much satisfaction. **The King thinks it is most creditable on the part of all those who have the management of the Fund that it should have been placed on such a sound foundation, and that its progress during the past year should have been so excellent."** Their Majesties have from the very first always displayed the deepest personal interest in this organisation of women workers. (Cheers.) You must remember that we owe a great deal to the King and Queen—you nurses especially—for there is no doubt that the courage of their Majesties—and it was really an act of courage which they displayed in identifying themselves with the Fund in the very earliest days as Patron and President—has had a most material influence on its success. We, of course, and all who are connected with the Fund have done our best, but I think on an occasion like this, when, for the first time, we have had the opportunity of hearing a letter read from the

King, which proves that he takes a live, a real, an individual interest in our Fund, it is well we should remember and should heartily appreciate that we really do owe very much to their Majesties. (Cheers.)

### Its Advantages to Nurses.

Mr. Thomas Charles Dewey, of the Prudential Insurance Company, in seconding the adoption of the report, congratulated the Fund on its prosperous condition and said: My only regret is that the nurses do not avail themselves to a larger extent of the unmistakable advantages—far beyond those of any other institution—which are afforded to them by the Royal National Pension Fund; and I should like, if possible, that those advantages should be put before the nurses in every hospital in the kingdom. The result of the voting for the Annuitants and Policy-holders was then announced.

### Policy-holders' Representatives.

The Chairman: I am sure we are very much indebted to the scrutineers for giving the time to go through all these cards. It takes them a long time, and they do it regularly, and we are really much indebted to them. In accordance with the ordinary practice I have to direct that the voting cards be destroyed. You notice that Mr. Gibbs said that if you want a new candidate you must nominate others than those who appear in this year's report before the end of January. I make that announcement now because the Council will, no doubt, take up the report—the important report—of the scrutineers. There is a financial aspect to it, and if we can save £30 a year, we shall save an annuity for one or possibly be able to give assistance to two nurses through the Benevolent Fund.

### Profit on Investments.

Mr. A. J. Waley: Mr. Chairman, ladies, and gentlemen, I have no hesitation, in reply to the invitation of Sir Henry Burdett, in making a few remarks as to your investments, but at this late hour I shall compress what I have to say as much as possible. Last year, in referring to the investments, I had to point out that there was a depreciation of £2,500 in the cost value of the invested funds. I am happy to say that the conditions have entirely changed, and this year the cost price of your invested funds was £900,044 5s. 1d., while the actual value was £915,332 12s. 7d., which shows a net profit of £15,288 7s. 6d.

The Chairman: What date is that?

Mr. Waley: I was going to refer to that. That is the valuation made by my firm at the end of December last. The gross profit shown then was £23,657 0s. 2d., against which had to be written off a depreciation of £8,368 12s. 8d., the major part of which—in fact, with the exception of £500, the whole of which was a depreciation—was shown in the value of British Government securities and English railway stocks. As you are, no doubt, well aware, the conditions of the money market have greatly changed in the last two months, and with the rise which has taken place in British Government funds and English railways since then, I think I can fairly say that nearly £5,000 of that depreciation has already come back, and that therefore the gross surplus shown over the cost price is practically £20,000. (Cheers.) Sir Henry Burdett and Mr. Dewey have referred as a satisfactory feature to the fact that the average rate of interest yielded by the investments is £4 1s. 3d. per cent., but it must not be forgotten that the period of depression through which the City has been passing in the last two or three years has been, perhaps, favourable to lenders, but unfavourable to borrowers. By that I mean to say that the Council have had exceptional opportunities during that period for investing your money under very favourable conditions. Now that the money market has emerged from the depression of the last few years there will be increasing difficulty in finding securities to yield that return, but I think you may feel every confidence that your Council will not in any way depart from the very wise principle which guides them of only seeking first-class investments; and you may have every confidence that the Fund will be administered and the investments chosen with the same amount of care which has always been shown in the administration of this Fund. I think you can show no better proof of the confidence you have in the Council than by re-electing the following gentlemen, who are retiring by rotation, and whose re-election I propose. They are Mr. R. Ernest Alexander, Mr. T. C. Dewey, the Hon. Egremont J. Mills, Mr. J. Pierpont Morgan, jun., and Mr. Archibald Williamson.

The retiring members of the Council were re-elected, likewise the auditors.

### Thanks to a Policy-holder.

After putting the last motion the Chairman said: I have spoken to you to-day chiefly on the subject of thrift and the importance of co-operation. I have now great pleasure in moving a vote of thanks to a lady who has rendered very material assistance to this Fund, being a policy-holder herself. Miss M. D. Brinton has written this pamphlet and it has had a very great success. It is written by a policy-holder and is entitled "The Pension Fund." By an Inquiring Policy-holder. It gives a full account of the Fund. It gives these nurses the reasons why it is desirable that they should join it, what is likely to happen to their money if they do, and from first to last it is a most interesting production. It therefore seems to me that it would be a good thing to thank Miss Brinton, and to thank her very warmly, for having written this pamphlet, and shown by her act how practically a policy-holder can help the Fund.

A vote of thanks to Miss Brinton was carried unanimously, and then Dr. S. H. Habershon said: I have much pleasure in moving a vote of thanks to our chairman for so kindly presiding to-day. I think he must be very proud himself of the magnificent result of his initial labours and at the successful way in which this Pension Fund is proceeding year by year. I think it is necessary to say what a debt of gratitude every pensioner in this Fund owes to Sir Henry Burdett and his disinterested labours on their behalf. I think the reward comes to him from the satisfaction of knowing the splendid success of this movement. (Cheers.) The Chairman returned thanks, and the formal proceedings having terminated, the policy-holders present and some of the members of the Council adjourned to tea, which was provided.

### Everybody's Opinion.

[Correspondence on all subjects is invited, but we cannot in any way be responsible for the opinions expressed by our correspondents. No communication can be entertained if the name and address of the correspondent are not given as a guarantee of good faith, but not necessarily for publication. All correspondents should write on one side of the paper only.]

### VICTORIA HOSPITAL, BLACKPOOL.

Miss E. M. Edwards, matron of Chelsea Hospital for Women, writes:—I see announced in to-day's issue of The Hospital that "Miss Edith Brown, who has been appointed sister at Victoria Hospital, Blackpool, was trained at Burton-on-Trent Hospital, and has since been staff nurse at Chelsea Hospital for Women." This latter is an error, except in so far as Miss E. Brown came here September 11th, 1902, as "staff" or assistant nurse, and left October 11th, 1902.

### WOMEN INSPECTORS OF MIDWIVES.

"Live and Let Live" writes:—Replying to "L. M. B.'s' letter in your issue of February 18th, may I ask what are the sacrifices of hospital trained nurses compared with those of home trained nurses? Why should home trained nurses and midwives not be given the post of districts? They are the people who understand that work, they have had to learn it, and after six months their merit is tested for two years. In doing their work and gaining their knowledge, sometimes they are in cottages with no doctor near them, no comforts, or scarcely anything for use and convenience at hand, while the hospital trained nurses have every comfort, everything for use and convenience at hand, and in every emergency, sister and doctor to go to for help. Fancy the responsibility home trained nurses and midwives have to take upon themselves! With this responsibility and careful experience they must indeed be dull if they do not get wise and learn their work.

### TEN YEARS WAITING FOR AN ANNUITY.

Dr. A. Ogier Ward, hon. sec. of the Trained Nurses Annuity Fund, 73 Cheapside, E.C., writes: I am sure you would not intentionally injure this charity. But your Note has, unfortunately, the apparent effect of contrasting what a nurse might possibly have provided for herself with what she has provided through this society. At least that is the impression your Note conveyed to a friend of mine. This society only insists on an entrance fee to prevent nurses from thinking they need not exercise any self-denial during their early working years. This and this alone is insisted on, otherwise all the annuity is pure charity, and the £15 payable on entrance is less than one year's annuity. I entirely endorse your inference that every nurse who can ought to insure in the Royal National Pension Fund. But this society finds ever so many applicants who never could insure owing to family claims, etc., and others who have insured and have broken down and had to withdraw their savings. It is for these we plead; not for the stalwarts who ought to but do not insure."

[We thought that we made it very clear that this excellent fund is entitled to the support of the charitable. The pity is that there should be nurses who from want of prudence, or—as in the case quoted—from lack of opportunity, are compelled to ask for its assistance.—Ed. The Hospital.]

### TRAVEL NOTES AND QUERIES.

By our Travel Correspondent.

Venice for Two Days (A Countrywoman).—You surely cannot seriously mean to take that long and expensive journey to remain in Venice only two days. Write me again saying exactly how much money you feel you can spend and how long your holiday lasts. I shall then be able to advise you for the best. I do not think you could vary your route in the way you propose, but I will find out for you. The cheapest route second class return is viâ Dieppe, Paris, the St. Gothard and Milan, and comes, roughly speaking, to about £8 5s. You can get good accommodation in a non-fashionable hotel in Venice for 7 lire per day, that would come to £2 for a week. Let me hear further and probably we can strike out something between us to enable you to see more of the fairy city.

Rules in Regard to Correspondence for this Section.—All questioners must use a pseudonym for publication, but the communication must also bear the writer's own name and address as well, which will be regarded as confidential. All such communications to be addressed "Travel Correspondent, 'Nursing Section of *The Hospital*,' 28 Southampton Street, Strand." No charge will be made for inserting and answering questions in the inquiry column, and all will be answered in rotation as space permits. If an answer by letter is required, a stamped and addressed envelope must be enclosed, together with 2s. 6d., which fee will be devoted to the objects of "The Hospital" Convalescent Fund. Ten days must be allowed before an answer can be published.

### Appointments.

[No charge is made for announcements under this head, and we are always glad to receive, and publish, appointments. The information, to insure accuracy, should be sent from the nurses themselves, and we cannot undertake to correct official announcements which may happen to be inaccurate. It is essential that in all cases the school of training should be given.]

Colchester Hospital. —. Miss Edith Porter has been appointed night sister. She was trained at the Western Infirmary, Glasgow. She has since been charge nurse of the Outdoor Department of the Children's Hospital, Sheffield, charge nurse at Friedenheim, London, a member of the staff of the Carlisle and North of England Trained Nurses' Home, and sister-in-charge of "the Branch Home" of that institution at Penrith.

Hackney Union Workhouse.—Miss Clara Neale has been appointed staff nurse. She was trained at the Edmonton Union Infirmary, and has since been staff nurse at the North-Eastern Hospital, London.

Hospital for Infectious Cases, Birkdale. — Miss Hughes has been appointed staff nurse. She was trained at the Salford Union Infirmary, and has since been nurse at Leeds Infectious Hospital.

Lodge Moor Hospital, Sheffield.—Miss Frances Pritchard has been appointed assistant matron. She was trained at Fir Vale Infirmary, Sheffield, where she was afterwards sister. She has also been charge nurse at the South-Eastern Hospital, London, has been attached to the Brooklyn Institute, Anerley, London, and night superintendent at Lodge Moor Hospital, Sheffield.

Manchester Children's Hospital, Pendlebury.—Miss Flora B. Cameron has been appointed lady superintendent. She was trained at the Western Infirmary, Glasgow, and has since been night superintendent and assistant matron at the Royal Infirmary, Bradford, and matron at the Children's Hospital, Bradford.

Nutford and Launditch Workhouse Infirmary.—Miss Florence Gane has been appointed superintendent nurse. She was trained at Norfolk and Norwich Hospital and has since been attached to the private staff of that institution.

St. Mary (Islington) Infirmary, Highgate Hill.—Miss Ada M. Boycott has been appointed sister. She was trained at the Metropolitan Hospital, Kingsland Road, London, where she has since been holiday sister.

Southwark Infirmary.—Miss Nina Whelpton Johnson has been appointed sister. She was trained at the Croydon General Hospital, and has since been staff nurse at the Maternity District Home, Plaistow. She holds the L.O.S. certificate.

Stafford General Infirmary.—Miss Ada Woolrich has been appointed sister. She was trained at Nottingham General Hospital, where she has since been staff nurse and theatre nurse.

Workhouse Infirmary, Gravelly Hill, Birmingham.—Miss Frances H. Brettell has been appointed home sister. She was trained at Mill Road Infirmary, Liverpool, and was afterwards charge nurse at Gore Farm Fever Hospital, Darenth, and at the Eastern Hospital, Homerton. She has also done private nursing, and has since been sister and night superintendent at the West Ham Infirmary, London.

### Echoes from the Outside World.

### Movements of Royalty.

THE 42nd anniversary of the marriage of the King and Queen was celebrated on Friday. Their Majesties spent the day quietly at Buckingham Palace until evening, when they drove to Marlborough House. They received numerous congratulatory messages from foreign Courts and other sources. On Tuesday afternoon the King held a Council, and the Queen left England on the morning of the same day to visit the King and Queen of Portugal, the King accompanying her from Buckingham Palace to Victoria Station. She was accompanied by Princess Victoria, and by Prince and Princess Charles of Denmark. The Royal party were detained at Portsmouth for the night on board the *Victoria and Albert* owing to the stormy weather. It is expected that her Majesty will be away from England for six weeks at least.

### The Capture of Mukden.

ON Friday Marshal Oyama telegraphed to Tokio, "To-day at 10 A.M. we occupied Mukden. An enveloping movement, which has been proceeding for several days, has completely attained its object. Fierce engagements are now in progress at various places near Mukden. We have taken an exceedingly large number of prisoners, and quantities of arms, ammunition, prisoners, fodder, and war material, but it has been impossible yet to count them." Thus, laconically, is summed up the story of the most sanguinary battle in history. Never before have 700,000 trained soldiers met in conflict and fought out their quarrel to the death, and the battle also constitutes a record because of the length of time it lasted. Mukden, the city now occupied by the Japanese, is about 400 miles from Peking, and nearly 300 from Port Arthur. The city is double-walled and is in the middle of a great alluvial plain more than 300 feet above the sea level. The population exceeds a quarter of a million, and the inhabitants are chiefly Chinese. Russian troops have occupied Mukden since October 1903, and since then great military buildings have been erected. Naturally, its capture by the Japanese has produced a profound impression in China, as well as in other parts of the world. General Kuropatkin, who has escaped with the remnants of his army to Tie-ling, is said to have asked permission of the Tsar to resign his command. The official despatches record the loss by the Russians of nearly 500 guns, a score of regimental colours, and over 200,000 men. On Wednesday an announcement bearing upon the future prosecution of the war was made in telegrams from Paris. It appears that the issue of the propcsed Russian loan for £24,000,000 has been indefinitely postponed.

### The Reconstruction of the Cabinet.

No fewer than four changes in the Cabinet were officially announced on Monday. Mr. Walter Long, President of the Local Government Board since 1900, has become Secretary for Ireland; Mr. Gerald Balfour, brother to the Prime Minister, President of the Board of Trade since 1900, is now President of the Local Government Board; Lord Salisbury, Lord Privy Seal for two years, succeeds Mr. Gerald Balfour at the Board of Trade; and Mr. Ailwyn Fellowes, a Junior Lord of the Treasury since 1900, enters the Cabinet for the first time as President of the Board of Agriculture. Mr. Gerald Balfour is 54, Mr. Long 51, Mr. Fellowes 50, and Lord Salisbury 42.

### New Ambassador at Vienna.

THE King has approved the appointment of Sir Edward Goschen as Ambassador to the Court at Vienna, in succession to Sir Francis Plunkett. Sir Edward is a younger brother of Lord Goschen. Born in 1847, he became an attaché in 1869, and has spent the whole of his life since in the Diplomatic Service. His appointment in 1884 to be Secretary of Legation at Peking was followed by his transfer to Copenhagen and Lisbon, where, in both cases, he acted for a time as Chargé de Affaires. He has subsequently been Secretary of Embassy at Washington, Minister Plenipotentiary at St. Petersburg and at Belgrade, and Minister at Copenhagen.

### Colliery Disaster in Wales.

ON Saturday morning another terrible colliery explosion was reported from South Wales. It occurred on Friday evening at the Cambrian Colliery, Clydach Vale, near Cardiff, one of the largest mining concerns in the Principality, nearly 4,000 men being employed by the owners. The noise caused by the explosion was terrific, and, in spite of drenching rain, thousands of people in the Rhondda Valley soon made their way up the hills to the scene of the catastrophe. There, it was found that the explosion had taken place at No. 1 down-shaft, and a relief gang was at once organised, consisting of the agent, the two managers, and the head mechanic. At the first there was no hope of any of the men in the pit being saved, but some of the injured, while seriously hurt, are likely to recover. It is feared that there are 26 killed, and nearly a score of horses were found dead in one of the stables. Clydach Vale Colliery is the highest in the Welsh coal-field, being 1,000 feet above sea level, it is lighted by electricity, is generally regarded as one of the finest-equipped collieries in South Wales, and has never before been the scene of an underground explosion.

### Royal Amateur Art Society.

LAST week Princess Louise Augusta, of Schleswig-Holstein, opened the Annual Exhibition of the Royal Amateur Art Society, which, by permission of Lord Howard de Walden, is held this year in Seaford House, Belgrave Square. As on previous occasions, the proceeds are divided among the Parochial Union Women Fund, the East London Nursing Society, and the Deptford Fund. The winners of the prizes included Mrs. J. Jardine, the Hon. Marian de Saumarez, Mrs. Benyon, and Mrs. Bethune. The collection, though necessarily on a small scale, was very representative, and included a fine head of Henry IV. of France, by E. Gautier Dagoty, lent by the King, dating from 1740; while the group devoted to paintings contained three flower studies by Princess Victoria Melita of Anemones, wall-flowers in a green glazed earthenware vase, and roses, which her Royal Highness permitted to be sold at moderate prices to help the objects of the exhibition. Miss Balfour, sister of the Prime Minister, sent a striking study of olive trees, and Lady Granby three portraits of children. The enamels were a very attractive section, and Mrs. Louis H. M. Dick was particularly successful in the designs and colouring of pendants, hatpins, and clasps, as well as in the ornamentation of combs of clear horn.

### Irish Central Bureau for Employment of Women.

THE first annual meeting of the Irish Central Bureau fo the Employment of Women has just been held in Dublin. Lady Dudley was in the chair, and Mrs. Humphry Ward moved the adoption of the report. The report is of a very exhaustive character, and shows that during the past year branches have been established at Belfast, Killarney, and Tralee. Four honorary secretaries for counties have been appointed, and the bureau has on its list the names of 341 applicants. The tables in the report indicate the great need of such an organisation and it appears that the bureau is daily proving increasingly useful to those it was founded to benefit.

### Notes and Queries.

**FOR REGULATIONS SEE PAGE 328.**

*Sanatorium.*

(178) Will you kindly inform me if there is a home or sanatorium where a lady in reduced circumstances suffering from phthisis can be received for a small charge ?—*Queen's Nurse.*

See the list of sanatoria, published by the National Association for the prevention of tuberculosis, obtainable from the Scientific Press, price 7d. with postage.

*Staff Nurse.*

(179) Can you tell me how to obtain an appointment as staff nurse, or, failing that, as assistant nurse in one of the large general hospitals in England or Scotland ? I hold a three years' certificate for general and fever training from one of the largest unions in the kingdom. My testimonials are good and I am otherwise fitted for the appointment.—*Staff Nurse.*

By answering advertisements, or by advertising yourself, in The Hospital.

*Children's Nurses.*

(180) Can you please tell me the best institution for training children's nurses ? Is there a Norland Hospital ? If so, could you kindly give me the address ?—*B. B. S.*

If you mean nurses for sick children, there are many children's hospitals where excellent training can be obtained. See "The Nursing Profession: How and Where to Train." There is no Norland Hospital, but the address of the Norland Institute, where children's nurses are trained, is Pembridge Square, London, W.

*Continental Nursing.*

(181) Will you be so kind as to let me know if there are any homes in London that send nurses to do "Continental Nursing," or if you could give me the names of any English homes on the Continent ?—*E. J.*

The English Nurses' Home, Villa Albany, Biarritz ; the Davos Invalids' Home, Davos Dorf, Switzerland ; the Association of Trained Nurses and Masseuses, 7 Via Rondinelli, Florence ; the Nice Nursing Institute, Villa Pilatte, Avenue Désambrois, Nice ; the Anglo-American Nursing Home, 265 Via Nomentana, Rome ; the Institute for Trained English Nurses, Sunny Bank, San Remo.

*Training.*

(182) In the interest of a young lady friend who contemplates training as a nurse, will you kindly tell me if the ——— Hospital is an authorised hospital really suitable for turning out thoroughly well-qualified nurses, and would the certificate be acceptable anywhere ?—*Nurse E. C.*

A certificate from a larger hospital would be more valuable. See "The Nursing Profession : How and Where to Train," which will give you full information.

I wish very much to become a nurse. Could I go as a probationer ? I am a servant now. Do you think any one would take me without any premium ?—*A. W.*

The Middlesex Hospital might particularly suit your case. Write to the Lady Superintendent, Mortimer Street, W.

*Army Nursing Service.*

(183) I should be obliged if you could inform me who the ladies are on the committee for selecting nurses for the Royal Army Nursing Service.—*J. M.*

The Nursing Board under which the Service is controlled is composed as follows :—President, vice-president, chairman, together with two members of the Advisory Board—Sir F. Treves, Bart., and the Director-General ; matron-in-chief, Miss Sidney Browne, R.R.C. ; two matrons of civil hospitals—Miss Monk and Miss Cave ; a representative of the India Office ; two members nominated by her Majesty — Viscountess Downe and the Hon. Sydney Holland.

*X-rays.*

(184) Will you kindly tell me if by means of the $x$-rays the position of the internal organs can be seen ? or any abnormality ?—*Nurse A.*

We have not sufficient space for a full reply to these questions.

**Handbooks for Nurses.**

| | Post Free. |
|---|---|
| "How to Become a Nurse : How and Where to Train." ... | 2s. 4d. |
| "The Nurses' Dictionary" (Pronouncing)... ... ... | 2s. 0d. |
| "Nursing : its Theory and Practice" (Lewis.) ... ... | 3s. 6d. |
| "The Light Treatment" (just published).... ... ... | 2s. 6d. |
| "A Complete Handbook of Midwifery." (Watson). ... | 6s. 4d. |

Of all booksellers or of The Scientific Press, Limited, 28 & 29 Southampton Street, Strand, London, W.C.

### For Reading to the Sick.

THE CONSOLER.

O Comforter of God's redeemed,
Whom the world does not see,
What hand should pluck me from the flood,
That casts my soul on Thee ?
Who would not suffer pain like mine,
To be consoled like me ?

Thy love has many a lighted path
No outward eye can trace,
And my heart sees Thee in the deep,
With darkness on its face,
And communes with Thee, 'mid the storm,
As in a secret place.

Deep unto deep may call, but I
With peaceful heart will say—
Thy loving-kindness hath a charge
No waves can take away ;
And let the storm that speeds me home
Deal with me as it may.

*A. F. Waring.*

---

He is leading us in paths which we have not known. Could we have ordered our own lot, it would not have been thus with us: we should have chosen a smoother road and an easier life. We should have gathered all our resources of happiness about us, all our treasures should have been kept within our reach. We should have spared ourselves all pain and sickness, and never should have known the bitterness of partings, and the throes of the mourner's anguish. But have we appealed from ourselves to Christ, from our own ignorance of what is really best for us, from our softness and self-indulgence, from our utter incapacity to direct ourselves, to Him the leader and guide of His people ?

---

Have we said, Lord, Thou knowest that if it were given me to choose my own path, and order my own future, I would bring that dangerous gift, and place it in Thy hands, and would say, "Choose Thou for me" ? If it be thus with us, and if we approve such a spirit of humility and distrust of ourselves as this, let us not shrink from the discipline of the life He is appointing for us. Let us take up the cross He lays upon us, and follow Him.

---

And the same might that chases away the shades of ignorance, doubt, and misbelief, shall be put forth to dispel the clouds of affliction, when He is satisfied that it has delivered its message to our spirit and accomplished its work. He will make darkness light before us, and crooked things straight.

The sooner we acquiesce in the wisdom, and acknowledge the love which sends the sorrow, the sooner its clouds shall be rolled away, and our path, that seemed to pass through a gloomy labyrinth out of which there was no issue, shall be made straight before our eyes; and its every ruggedness shall be smoothed, and we shall acknowledge that His ways are the ways of pleasantness and all his paths are peace.

*T. V. Fosberry.*

